# Identification of Active Components in *Connarus ruber* Extract Exhibiting Anti-Glycation Effects

**DOI:** 10.3390/medicines12040029

**Published:** 2025-12-03

**Authors:** Ryoji Taniguchi, Ryusuke Nakatsuka, Yuka Sasaki, Mariko Takenokuchi, Takashi Maoka, Tomio Iseki, Hirohito Kubo, Tadashige Nozaki

**Affiliations:** 1 Department of Pharmacology, Faculty of Dentistry, Osaka Dental University, 8-1 Kuzuhahanazono-cho, Hirakata 573-1121, Osaka, Japan; nakatsuka-r@cc.osaka-dent.ac.jp (R.N.); sasaki-y@cc.osaka-dent.ac.jp (Y.S.); nozaki@cc.osaka-dent.ac.jp (T.N.); 2 Faculty of Pharmacological Sciences, Daiichi University of Pharmacy, 22-1 Tamagawa-machi, Minami-ku, Fukuoka-shi 815-8511, Fukuoka, Japan; m-takenokuchi@daiichi-cps.ac.jp; 3 Division of Food Function and Chemistry, Research Institute for Production Development, Morimoto-cho, Shimogamo, Sakyo-ku, Kyoto-shi 606-0805, Kyoto, Japan; maoka@mbox.kyoto-inet.or.jp; 4 First Department of Oral and Maxillofacial Surgery, Faculty of Dentistry, Osaka Dental University, 8-1 Kuzuhahanazono-cho, Hirakata 573-1121, Osaka, Japan; iseki@cc.osaka-dent.ac.jp (T.I.); kubo-h@cc.osaka-dent.ac.jp (H.K.)

**Keywords:** *Connarus ruber*, Connaraceae, epicatechin, procyanidin A2, dental pulp stem cells, glycation

## Abstract

Background: Glycation, a non-enzymatic reaction between sugars and biomolecules, leads to the formation of advanced glycation end-products (AGEs), which are implicated in the progression of chronic diseases. *Connarus ruber* (Poepp.) Planch (*C. ruber*), a traditional medicinal plant used for diabetes, has shown anti-glycation activity. This study aimed to identify the active components in *C. ruber* extract and elucidate their anti-glycation mechanisms. Methods: Using NMR and LC-MS analyses, we identified epicatechin and procyanidin A2 as major polyphenolic constituents. Collagen glycation assays were performed to evaluate the inhibitory effects of these compounds on fructose- and glyceraldehyde (GA)-induced glycation. Additionally, their cytoprotective effects were assessed using GA-induced cytotoxicity assays in dental pulp stem cells (DPSCs). Results: Both epicatechin and procyanidin A2 inhibited fructose- and GA-induced glycation in a dose-dependent manner, showing greater efficacy than aminoguanidine. Furthermore, these compounds significantly alleviated GA-induced cytotoxicity in DPSCs. Conclusions: These findings suggest that epicatechin and procyanidin A2 are candidate contributors to the anti-glycation and cytoprotective effects of *C. ruber*. The results support the potential of *C. ruber* extract as a source of therapeutic agents for glycation-related diseases and for enhancing stem cell viability.

## 1. Introduction

Sugars and their derivatives are essential nutrients for human cells, supporting various physiological functions. However, certain reducing sugars, such as fructose and glucose can induce glycation, a non-enzymatic process that occurs during sugar metabolism [1]. Glycation modifies lysine and arginine residues of proteins, leading to the formation of advanced glycation end products (AGEs) [2,3,4]. AGEs play a key role in the pathogenesis of various chronic diseases, including diabetes, myocardial infarction, stroke, Alzheimer’s disease, osteoporosis, and malignancies [5,6,7,8,9]. The accumulation of AGEs has been suggested to reduce tissue elasticity by promoting the cross-linking of collagen molecules in the extracellular matrix [10]. Tissues rich in collagen, such as the skin, skeletal muscle, tendons, heart, and lens, are particularly vulnerable to the detrimental effects of AGE accumulation. The functional decline observed in these tissues with aging is closely linked to the accumulation of AGEs [6,9,11,12]. Cellular dysfunction occurs not only through the cross-linking of intracellularly accumulated AGEs with proteins, lipids, and DNA but also through the binding of extracellular AGEs to the receptor for advanced glycation end products (RAGE) on the cell membrane. This interaction generates reactive oxygen species (ROS), which subsequently induce the production of pro-inflammatory mediators, thereby amplifying the inflammatory response [7,13,14].

To mitigate glycation-induced toxicities, the primary strategy has traditionally focused on reducing blood glucose levels. Management of blood glucose levels can be achieved through dietary modifications, exercise interventions, and pharmacotherapy. Pharmacological treatments work by enhancing insulin secretion, promoting glucose uptake in adipose and muscle tissues, inhibiting glucose absorption in the small intestine, suppressing hepatic gluconeogenesis, or increasing renal glucose excretion. However, all these treatment modalities carry the risk of hypoglycemia, which is a significant acute complication of diabetes. Recently, efforts have been made to explore anti-glycation agents that directly modulate glycation and suppress AGE accumulation. Aminoguanidine (AG) and alagebrium (ALT711) have been studied as representative anti-glycation agents. AG reacts with intermediates such as carbonyl compounds, thereby inhibiting AGE formation. Although clinical trials demonstrated that AG could reduce diabetic complications, side effects including liver dysfunction, gastrointestinal disturbances, and anemia were observed. As a result, AG has not been approved for clinical use [15,16]. On the other hand, ALT711 has the ability to cleave AGE-mediated protein cross-links. Experiments using animals have demonstrated that ALT711 reverses arterial and ventricular wall stiffness in healthy older rhesus monkeys, a randomized human clinical trial during a period of one year, however, showed no significant effect on arterial stiffness and ALT711 has also not been approved for clinical use [15,17].

Following the failure of AG and ALT711 to reach clinical application, research has increasingly focused on food-derived antiglycation agents. In the Amazon, a traditional remedy known as ‘miraruíra’ (cipó–miraruíra) has long been used for diabetes and related metabolic complaints, and recent DI–MS chemoprofiling of marketed “miraruíra” identifies *Connarus ruber* (Poepp.) Planch. (*C. ruber*) as a principal botanical source [18]. *C. ruber* is a plant of the Connaraceae family, and is native to the South American Amazon. We previously showed that *C. ruber* extract inhibits collagen glycation in vitro and reduces AGE-positive mesangial cells in the renal glomeruli of streptozotocin-induced diabetic rats in vivo [19]. However, the active constituents and mechanisms of action of *C. ruber* remain unclear.

The oral cavity, characterized by tissues rich in collagen, is one of the areas most exposed to sugar, making it particularly susceptible to glycation. Common oral manifestations in diabetic patients include dental caries, periodontal disease, salivary gland disorders (xerostomia), mucositis, taste disturbances, increased susceptibility to infections, and delayed wound healing. These oral complications occur more frequently and progress more rapidly in individuals with poor glycemic control, suggesting a significant association between glycation and these oral symptoms.

Dental pulp stem cells (DPSCs), a subset of mesenchymal stem cells (MSCs) derived from the neural crest, exhibit the ability to regenerate both connective and nervous tissues. Currently, they are being investigated for various regenerative applications, including bone regeneration, salivary gland repair, tooth regeneration, and dental pulp restoration. DPSCs demonstrate significant differentiation potential into diverse cell types, such as osteoblasts and neural cells, highlighting their therapeutic value in the treatment of oral dysfunctions and tissue repair.

AGEs can be classified immunologically into approximately seven types, depending on differences in metabolic pathways and intermediates involved. Among these, AGEs derived from glyceraldehyde (GA) are particularly toxic and are referred to as toxic AGEs (TAGEs). TAGEs are considered to be produced from the metabolism of starches found in staples like rice, bread, and noodles, as well as from the breakdown of sugars such as sucrose and high-fructose corn syrup, which are commonly added to beverages and processed foods. Notably, fluctuations in TAGE levels are closely associated with dietary habits in humans [20]. Given their toxicity, GA has become one of the focal points in research aimed at elucidating glycation’s role in disease progression and identifying potential therapeutic targets to mitigate their harmful effects.

In this study, we performed a component analysis of *C. ruber* and found that it contains several polyphenols. Among these, epicatechin and procyanidin A2 were identified as inhibitors of GA-induced collagen glycation and of GA-induced cytotoxicity in DPSCs.

## 2. Materials and Methods

### 2.1. Preparation of C. ruber Extract

*C. ruber* harvested in the Amazon with the permission of Instituto Nacional de Pesquisas da Amazônia (INPA) was purchased from Agrorisa Industria e Comercio de Produtos Alimenticios Naturais Ltda. (Manaus, Brazil). Four grams of shredded *C. ruber* stems were extracted with 80 mL of water at 80 °C for 30 min. The extraction was filtered and sterilized using a 0.22 µm vacuum-driven filtration system (IWAKI, Tokyo, Japan) and stored at 4 °C until use. For component analysis, the sterilized extract was lyophilized.

### 2.2. Preparation of Human Dental Pulp Stem Cells (DPSCs)

Human DPSCs (adult third molar-derived; Lonza, Basel, Switzerland) were purchased. The cells were cultured in basal medium consisting of Dulbecco’s modified Eagle’s medium, high glucose (DMEM; Nacalai Tesque, Kyoto, Japan) supplemented with 10% heat-inactivated fetal bovine serum (FBS) and 1% penicillin-streptomycin mixed solution (Nacalai Tesque, Kyoto, Japan). Cells were grown at 37 °C in a humidified 5% CO_2_ atmosphere and were used between passages 6–9.

### 2.3. Collagen Glycation Assays

The in vitro anti-glycation activities of *C. ruber* extract, epicatechin (FUJIFILM Wako Pure Chemical, Osaka, Japan), and procyanidin A2 (Adipogen Life Sciences, San Diego, CA, USA) were evaluated using a collagen glycation assay kit (Cosmo Bio, Tokyo, Japan) according to the manufacturer’s instructions. Sterilized *C. ruber* extract (0.03%, 0.15%, 0.25%), epicatechin (0, 0.8, 4, 20 mM), and procyanidin A2 (0, 0.8, 4, 20 mM) were prepared as test solutions.

GA-induced glycation assay: Forty microliters of each test solution and 10 μL of 500 mM glyceraldehyde (GA) solution were added to collagen gel-coated 96-well plates. After incubation at 37 °C in a humidified chamber for 24 h, fluorescence was measured at an excitation wavelength of 370 nm and an emission wavelength of 440 nm using a microplate spectrometer (SpectraMax M50S, Molecular Devices, Tokyo, Japan).

Fructose-induced glycation assay: In a separate set of wells, 10 μL of each test solution and 50 μL of 200 mM fructose solution were added to collagen gel-coated 96-well plates. Plates were incubated at 37 °C in a humidified chamber, and fluorescence (λ_ex = 370 nm, λ_em = 440 nm) was measured after 1, 2, 3, and 4 weeks.

For both assays, changes in fluorescence were calculated as ΔF(t) = F(t) − F(0), where F(0) is the fluorescence at the start of incubation.

### 2.4. Cytotoxicity Analysis

Cytotoxicity analysis was performed using Cell Counting Kit-8 (CCK-8) (Dojindo Molecular Technologies, Kumamoto, Japan) according to the manufacturer’s instructions. Briefly, DPSCs were seeded on 96-well plates at a density of 5 × 10^3^ cells/well and cultured for 24 h at 37 °C. Viability of cells, treated with GA (1 mM, 2 mM, 4 mM) for 24 h, was measured by CCK-8 assays. Absorbance was measured at 470 nm on a SpectraMax M50S.

### 2.5. Inhibition of GA-Induced Cytotoxicity

Cell viability was evaluated by trypan blue staining. DPSCs were seeded on 6-well plates at a density of 8.5 × 10^4^ cells/well and cultured for 24 h at 37 °C. Cells were then treated with epicatechin (1 nM, 10 nM, 100 nM and 1000 nM) or procyanidin A2 (1 nM, 10 nM, 100 nM and 1000 nM), and 1 mM GA for 24 h. Cells were then dissociated in 0.25% Trypsin/0.53 mM EDTA solution (Nacalai Tesque, Kyoto, Japan) and stained using 0.4% trypan blue solution (Nacalai Tesque, Kyoto, Japan). Live cells were counted, and cell viability was calculated. Cell viability (%) was normalized to the GA (–) condition at the corresponding time point, which was set to 100%.

### 2.6. Nuclear Magnetic Resonance (NMR) Spectroscopy and Liquid Chromatography Analysis

Lyophilized *C. ruber* extract samples were reconstituted in dimethyl sulfoxide (DMSO) and used for structural analysis. 1H NMR (500 MHz) and 13C NMR (125 MHz) spectra were generated with a Varian UNITY INOVA 500 spectrometer (Agilent Technologies, Santa Clara, CA, USA). LC (liquid chromatography)/DAD (diode array detection)/MS analysis was performed with ACQUITY Ultra Performance Liquid Chromatography System (Waters, Tokyo, Japan) equipped with Xexo G2-Q TOF MS spectrometer (Waters, Tokyo, Japan). LC/DAD/MS analyses were performed following conditions; Detection ESI Positive or Negative ion mode; Column: ACQUITY UPLC C18 1.7 μm 2.1 i.d. ×100 mm, Mobile phase: acetonitrile: water (5:95) to acetonitrile (100), 0 to 10 min linear gradient, Flow rate: 0.3 mL/min.

### 2.7. Statistical Analysis

The data were evaluated by one-way ANOVA with Tukey’s multiple comparison procedure between control and experimental groups. *: *p* < 0.05, **: *p* < 0.01, ***: *p* < 0.001.

## 3. Results

### 3.1. C. ruber Extract Inhibits Collagen Glycation Induced by Fructose and Glyceraldehyde (GA)

**Figure 1:**
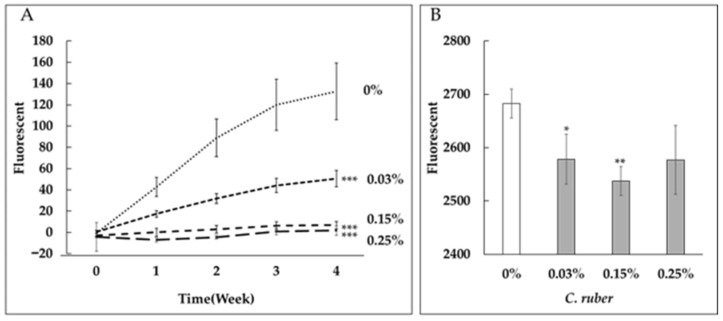
Dose-dependent inhibition of collagen glycation by *C. ruber* extract. Collagen glycation assays were performed to evaluate the anti-glycation activity of *C. ruber* extract at concentrations of 0.03%, 0.15%, and 0.25%. (**A**) Inhibition of fructose-induced collagen glycation. (**B**) Inhibition of glyceraldehyde (GA)-induced collagen glycation. Fluorescence intensity was measured at λex = 370 nm and λem = 440 nm after 24 h (GA) or weekly for 4 weeks (fructose). *C. ruber* extract demonstrated significant, dose-dependent inhibition of collagen glycation in both models. Each line represents the mean ± SD of four independent measurements. *: *p* < 0.05, **: *p* < 0.01, ***: *p* < 0.001.

We first evaluated the anti-glycation activity of *C. ruber* extract by conducting collagen glycation assays. *C. ruber* extract inhibited both fructose- and glyceraldehyde (GA)-induced collagen glycation in a dose-dependent manner (Figure 1).

### 3.2. Structural Analysis of Compounds in C. ruber Extract

**Figure 2:**
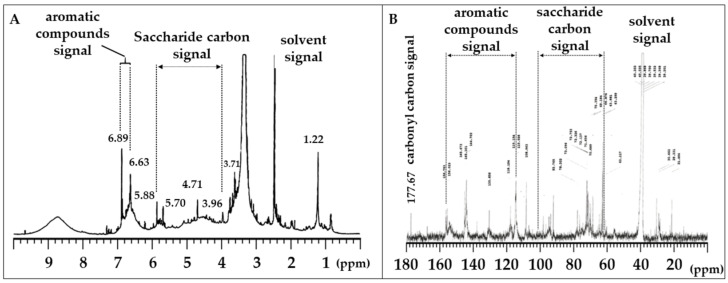
^1^H NMR (**A**) and ^13^C NMR (**B**) spectra of lyophilized *C. ruber* extract. NMR spectra were recorded in dimethyl sulfoxide-d_6_. In the ^13^C NMR spectrum, saccharide carbon signals (63–102 ppm), aromatic carbon signals (115–156 ppm), and a carbonyl carbon signal (177.67 ppm) were observed.

To explore anti-glycation compounds in *C. ruber* extract, we applied NMR analysis. Lyophilized *C. ruber* extracts were analyzed by 1H NMR (Figure 2A) and 13C NMR (Figure 2B). As shown in Figure 2A, chemical shift signals between 1.0 and 7.0 ppm were recognized in 1H NMR operated at 500 MHz. Among them, peaks from 3.96 to 5.88 ppm were presumed to be saccharide-derived signals (Figure 2A). In addition, signal peaks from 6.63 to 6.89 ppm were thought to be derived from aromatic compounds, such as polyphenols (Figure 2A). Moreover, 13C NMR analysis operated at 125 MHz confirmed numerous chemical shift signals from 20 to 180 ppm. Consistent with the 1H NMR analysis, saccharide-derived signals (63 to 102 ppm) and aromatic compound-derived signals (115 to 156 ppm) were recognized among the chemical shifts (Figure 2B). Besides these characteristic signals, a signal peak of an ester carbonyl carbon was observed at 177 ppm (Figure 2B). These results clearly demonstrated that *C. ruber* extract includes saccharides and phenols, including polyphenols.

### 3.3. Identification of Candidate Anti-Glycation Compounds in C. ruber Extract

**Figure 3:**
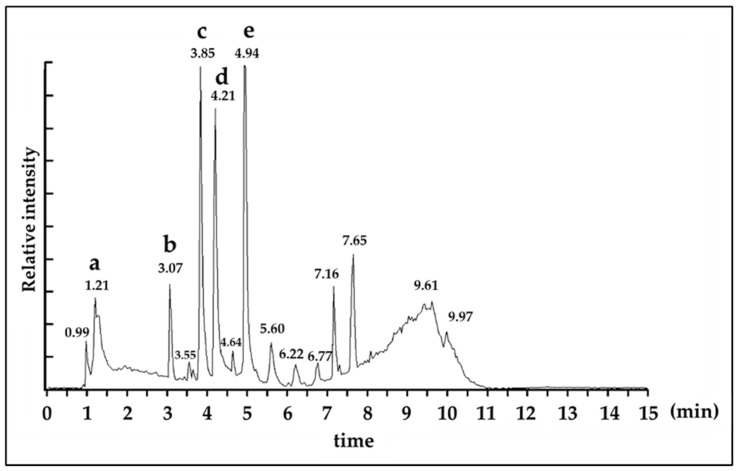
Total ion chromatogram (ESI positive ion mode) of *C. ruber* extract. Peak identification a: gallic acid, b: chlorogenic acid, c: procyanidin B, d: epicatechin, e: procyanidin A2. Details of identification data of each peak was described in Table 1. Detection: ESI Positive ion mode, Column: ACQUITY UPLC C18 1.7 μm 2.1 i.d. ×100 mm, Mobile phase: acetonitrile: water (5:95) to acetonitrile (100), 0 to 10 min linear gradient, Flow rate: 0.3 mL/min.

We then performed liquid chromatography-mass spectrometry (LC-MS) analysis to identify specific anti-glycation compounds in *C. ruber* extract. As shown in Figure 3, five distinctive metabolite-derived signal peaks were confirmed. Five metabolite peaks were eluted at 1.21 min (a), 3.07 min (b), 3.85 min (c), 4.21 min (d), 4.94 min (e) (Figure 3). These metabolites possessed an identical un-protonated molecular ion; [M−H]^−^ with gallic acid (*m*/*z* 169), and protonated molecular ion; [M+H]^+^ or [M+Na]^+^ with chlorogenic acid (*m*/*z* 355), procyanidin B (*m*/*z* 601 and 579), epicatechin (*m*/*z* 313 and 291) and procyanidin A2 (*m*/*z* 599 and 577), respectively (Figure 3 and Table 1).

### 3.4. Epicatechin and Procyanidin A2 Reduce Collagen Glycation

**Figure 4:**
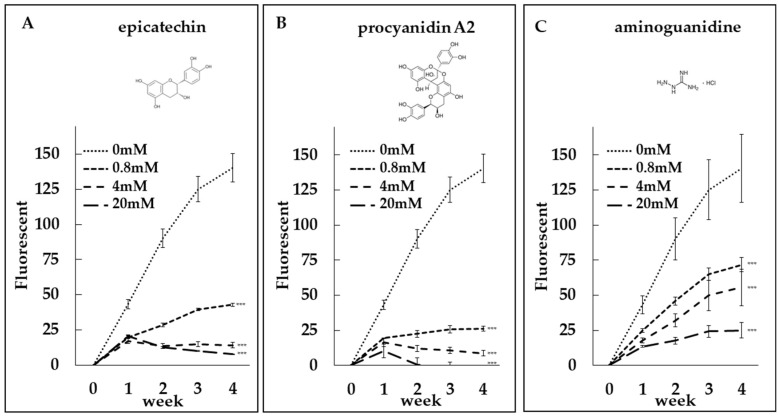
Dose-dependent anti-glycation effects of epicatechin and procyanidin A2. Collagen glycation assays were performed to evaluate the inhibition of fructose-induced collagen glycation by (**A**) epicatechin at concentrations of 0 mM, 0.8 mM, 4 mM, and 20 mM, (**B**) procyanidin A2 at concentrations of 0 mM, 0.8 mM, 4 mM, and 20 mM, and (**C**) aminoguanidine (AG) as a positive control. Fluorescence intensity was measured at λex = 370 nm and λem = 440 nm. Both epicatechin and procyanidin A2 demonstrated significant, dose-dependent inhibition of collagen glycation, comparable to the positive control. ***: *p* < 0.001.

**Figure 5:**
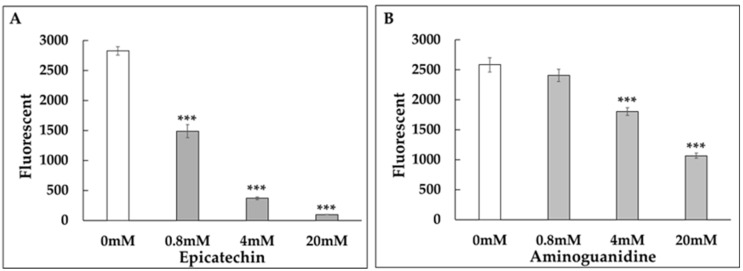
Dose-dependent inhibition of GA-induced collagen glycation by epicatechin and aminoguanidine (AG). Collagen glycation assays were conducted to evaluate the anti-glycation activity of (**A**) epicatechin at concentrations of 0 mM, 0.8 mM, 4 mM, and 20 mM and (**B**) AG as a positive control. Fluorescence intensity was measured at λex = 370 nm and λem = 440 nm after 24 h of incubation. Epicatechin demonstrated a stronger inhibition of collagen glycation compared to AG, in a dose-dependent manner. ***: *p* < 0.001.

To evaluate the anti-glycation potential of epicatechin and procyanidin A2, we conducted collagen glycation assays. Both compounds, along with aminoguanidine (AG), which served as a positive control, inhibited fructose-induced collagen glycation in a dose-dependent manner (Figure 4A–C). Additionally, epicatechin as well as AG also inhibited GA-induced collagen glycation in a dose-dependent manner (Figure 5A,B). Notably, the inhibitory potency of epicatechin was greater than that of AG.

### 3.5. GA-Induced Cytotoxicity

**Figure 6:**
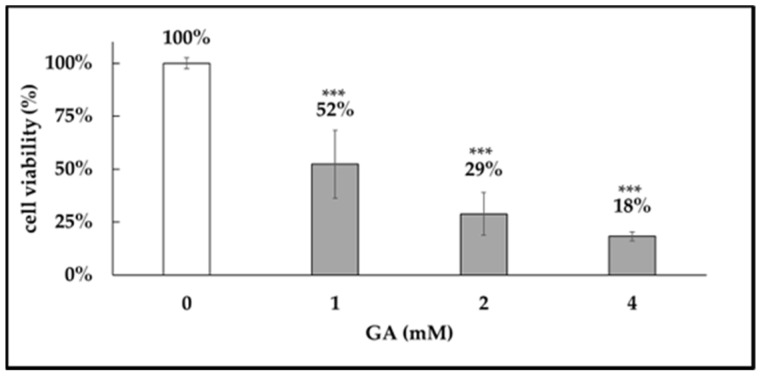
GA-induced cytotoxicity. Cytotoxic effects of glyceraldehyde (GA) on dental pulp stem cells (DPSCs). DPSCs were treated with GA at concentrations of 1 mM, 2 mM, and 4 mM for 24 h, and cell viability was assessed using the Cell Counting Kit-8 (CCK-8) assay. Absorbance was measured at 470 nm. GA exhibited dose-dependent cytotoxicity, with an LD50 of approximately 1 mM. ***: *p* < 0.001.

GA exhibited a dose-dependent cytotoxicity on dental pulp stem cells (DPSCs), with an LD50 of approximately 1 mM (Figure 6). Therefore, this concentration was used for subsequent experiments.

### 3.6. Epicatechin and Procyanidin A2 Inhibited GA-Induced Cytotoxicity

**Figure 7:**
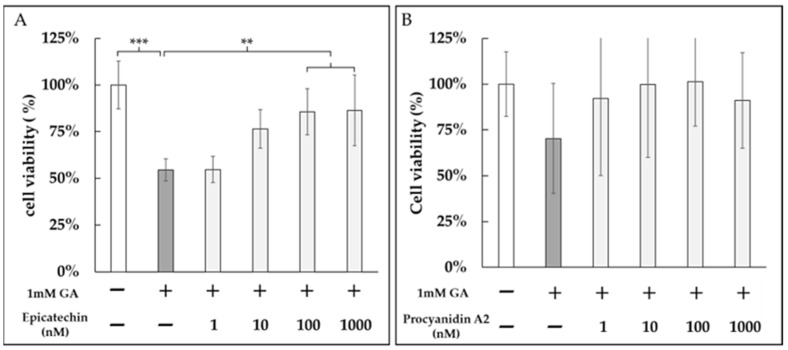
Protective effects of epicatechin and procyanidin A2 against GA-induced cytotoxicity in DPSCs. DPSCs were treated with 1 mM GA in the presence of (**A**) epicatechin (1 nM, 10 nM, 100 nM, and 1000 nM) or (**B**) procyanidin A2 (1 nM, 10 nM, 100 nM, and 1000 nM) for 24 h. Cell viability was assessed by trypan blue staining, and live cells were counted. Both epicatechin and procyanidin A2 significantly mitigated the reduction in cell viability caused by GA treatment. **: *p* < 0.01, ***: *p* < 0.001.

We next evaluated whether epicatechin and procyanidin A2 could protect against GA-induced cytotoxicity. Both epicatechin and procyanidin A2 treatments mitigated the reduction in cell viability caused by treatment with 1 mM GA (Figure 7A,B).

## 4. Discussion

Recently, we demonstrated that *C. ruber* extract inhibited the deposition of advanced glycation end-products (AGEs) in mesangial cells of diabetic rat models [19]. Additionally, it suppressed collagen glycation induced by fructose, reducing sugar (Figure 1A) [19] and glyceraldehyde (GA) (Figure 1B) in vitro. However, the precise mechanisms underlying the anti-glycation activity of *C. ruber* extract remain unclear.

Glycation is a non-enzymatic reaction between reducing sugars and the free amino groups of proteins, lipids, or nucleic acids. This process initiates with the formation of a reversible Schiff base, which is subsequently converted into a covalently bound Amadori product. As these intermediates undergo further complex reactions, irreversible advanced glycation end products (AGEs) are formed. These AGEs, along with their glycated intermediates, contribute to cellular and tissue damage, a phenomenon termed “glycation stress.” Additionally, reactive oxygen species (ROS) are generated during this process, exacerbating cellular damage [8]. The interaction of extracellular AGEs with the receptor for AGEs (RAGEs) on cell membranes further amplifies cellular damage and inflammation by stimulating ROS production within the cell. ROS also accelerates the formation of AGEs, a process known as “glycoxidation”, where glycation and oxidation reactions synergistically enhance cellular damage [21]. Glycation stress under hyperglycemic conditions, particularly when accelerated by glycoxidation plays a crucial role in the onset and progression of various diseases, including diabetes, cardiovascular diseases, neurodegenerative disorders like Alzheimer’s disease, osteoporosis, and even malignancies. Among the glycation intermediates, glyceraldehyde (GA) and methylglyoxal (MG) have been well studied and are shown to stimulate ROS production [7,9,20]. In our study, we found that *C. ruber* inhibited fructose-induced collagen glycation in a non-cellular system where no metals were present in the reaction mixture [19], suggesting a minimal role of oxidative processes. Additionally, *C. ruber* effectively protects against GA-induced cytotoxicity in dental pulp stem cells (DPSCs), and mitigates MG-induced cytotoxicity in HL60 cells [19,22], demonstrating its protective effects against glycation intermediates and oxidative damage. This observation indicates that *C. ruber* likely intervenes at the earliest stages of glycation, preventing the formation of the initial Schiff base and Amadori product. Furthermore, its efficacy in later stages of the glycation process, particularly where reactive oxygen species (ROS) play a role, underscores its dual action in inhibiting both glycation and oxidative damage (glycoxidation).

Glycation stress is significantly elevated in obese and diabetic patients. MSCs derived from these patients show reduced proliferation, differentiation, and immunomodulatory capabilities, indicating that glycation stress contributes to the deterioration of MSC quality [23]. DPSCs, a type of MSC possess the ability to differentiate into various tissues, including bone, cartilage, adipose tissue, nerves, and muscles [24,25,26,27], making them highly valuable for tissue repair and regenerative medicine [25,27]. In our study, we focused on DPSCs to analyze the effects of glycation stress and found that *C. ruber* extract significantly mitigated this cytotoxic effect of GA [22].

Unlike synthetic agents such as aminoguanidine (AG) and alagebrium (ALT711), which have faced clinical limitations due to adverse effects and limited efficacy [15,16,17], *C. ruber* has demonstrated safety and efficacy in animal models, showing no signs of hypoglycemia or other side effects [19]. Furthermore, its long history of traditional use among indigenous populations in the Amazon supports its safety in humans, with no reported adverse effects. These factors underscore the promise of *C. ruber* extract as a potent candidate for anti-glycation therapies.

To better understand the active components of *C. ruber* extract and their mechanisms, we conducted NMR spectroscopy (Figure 2A,B) and LC-MS (Figure 3), and identified several polyphenolic compounds, including gallic acid, chlorogenic acid, procyanidin B, epicatechin (EC), and procyanidin A2 (PA2) (Table 1). To our knowledge, among the identified constituents only EC has been reported as a component of *C. ruber* [18]. EC is also widely recognized as a free-radical-quenching antioxidant and has been proposed as a plausible antiglycation candidate in glycation-related contexts [28,29]. Accordingly, we first evaluated the anti-glycation activity of EC (Figure 4A) and PA2, A-type interflavan dimer of EC (Figure 4B).

Both compounds inhibited fructose-induced collagen glycation in a dose-dependent manner (Figure 4A,B). Notably, the inhibitory effects of epicatechin and procyanidin A2 were stronger than those of aminoguanidine (AG), a well-known positive control in glycation studies (Figure 4A–C). Epicatechin also showed significantly stronger inhibitory effects than AG on GA-induced collagen glycation (Figure 5A,B).

It has been suggested that the phenolic hydroxyl groups of polyphenols contribute to their antiglycation activity [28,29]. Epicatechin is a catechol-bearing flavanol with multiple phenolic hydroxyl groups, whereas procyanidin A2 is an A-type interflavan dimer (C–O–C linkage) with high phenolic density (Figure 4A,B).

Polyphenols can attenuate glycation through a range of routes, including (I) trapping of RCS; (II) inhibition of AGE formation; (III) blockade of AGE–RAGE interactions; (IV) inhibition of protein cross-linking (anti-cross-linking), with consequent protein protection; (V) suppression of ROS generation and antioxidant chain-breaking/redox modulation and (VI) chelation-mediated capture of catalytic metals that secondarily inhibits ROS generation [28,29].

Our collagen antiglycation evaluation system is cell-free and metal-free; therefore, the contributions of mechanisms such as AGE–RAGE interaction blockade, antioxidant chain-breaking/ROS suppression, and metal chelation to the observed antiglycation effects are expected to be minimal. The most parsimonious interpretation of the observed inhibition is RCS trapping (reduction in the GA pool), together with non-covalent protection of lysine/arginine residues and consequent cross-link inhibition. Furthermore, at higher concentrations, both epicatechin and procyanidin A2 exhibited a time-dependent reduction in fluorescence (Figure 4A,B), indicating that these compounds may not only inhibit the formation of fluorescent AGEs but also promote their breakdown. This dual action suggests their potential as both inhibitors and degraders of AGEs, offering a broader approach to mitigating glycation-related damage.

We next performed cell-based assays. EC significantly attenuated GA-induced cytotoxicity in DPSCs in a dose-dependent manner (Figure 7A). By contrast, PA2 produced a dose-graded reduction that did not reach statistical significance (Figure 7B). Although PA2, with higher density of phenolic hydroxyl groups, would be expected to confer greater reactivity and thus stronger antiglycation activity than EC, PA2 nonetheless yielded a weaker cytoprotective effect than EC in the DPSC assay. One plausible explanation is that monomeric EC (Figure 4A) diffuses to the pericellular space more readily, whereas PA2 non-covalently associates with proteins in the medium because of greater hydroxylation and multivalent binding capacity (Figure 4B). This would reduce its free fraction and the effective local concentration near cells. Consequently, at equivalent nominal dose, initial reaction rates and apparent cellular activity could favor EC.

These results suggest that attenuating GA-induced toxicity in DPSCs may enhance the overall effectiveness of regenerative medicine. Together with the cell-free data, these findings reaffirm the utility of *C. ruber* extract—which contains EC and PA2—as an antiglycation botanical, and further indicate that EC and PA2 can contribute to advances in glycation control and to the development of therapeutic interventions for glycation-related diseases.

## 5. Conclusions

In summary, we identified (−)-epicatechin (EC) and procyanidin A2 (PA2) in *Connarus ruber* extract and showed that both suppress collagen glycation in fructose- and GA-driven models and mitigate GA-induced cytotoxicity in DPSCs. Under cell-free, metal-free assay conditions, the most plausible mechanisms involve trapping of reactive carbonyl species and noncovalent protection of lysine/arginine residues. In the cell-free assay PA2 exhibited greater antiglycation potency, whereas in cells EC afforded stronger cytoprotection, indicating complementary, context-dependent strengths. Time-dependent fluorescence declines at higher concentrations are consistent with post-formation attenuation of fluorescent AGEs and raise the possibility of partial breaker-like activity, which warrants mechanistic confirmation. Taken together, *C. ruber* extract and its constituents EC and PA2 emerge as candidates for comprehensive glycation control and as promising adjuncts to preserve stem-cell function in regenerative medicine.

## Figures and Tables

**Table 1:** Identified compounds from *C. ruber* extract by ESI mass spectrometry.

Peak	Retention Time	Identification	Observed MS	Ion Formula	Calculated MS	Error	Fragment Ions	UV Max
	(min)		(*m*/*z*)			(ppm)	(*m*/*z*)	(nm)
a	1.21	Gallic acid	169.0144	[M−H]^−^ C_7_H_5_O_5_	169.0137	4.1		270
b	3.07	Chlorogenic acid	355.1083	[M+H]^+^ C_16_H_19_O_6_	355.1029	15.2	163	277
c	3.85	Procyanidin B2	601.1299	[M+Na]^+^ C_30_H_26_O_12_Na	601.1322	−3.8	579, 427, 409, 291	279
			579.1477	[M+H]^+^ C_30_H_27_O_12_	579.1503	−4.5		
d	4.21	Epicatechin	313.0709	[M+Na]^+^ C_15_H_14_O_6_	313.0688	6.7	207, 147, 139, 123	279
			291.0963	[M+H]^+^ C_15_H_15_O_6_	291.0869	32.3		
e	4.94	Procyanidin A2	599.1122	[M+Na]^+^ C_30_H_24_O_12_Na	599.1166	3.0	577, 437, 425, 287	279
			577.1353	[M+H]^+^ C_30_H_25_O_12_	577.1346	3.1		

## Data Availability

The data presented in this study are available on request from the corresponding author. The data are not publicly available due to privacy and ethical restrictions.

## Abbreviations

The following abbreviations are used in this manuscript:

AG	Aminoguanidine
AGEs	Advanced glycation end-products
ALT711	Alagebrium
CCK-8	Cell Counting Kit-8
*C. ruber*	*Connarus ruber*
DPSCs	Dental pulp stem cells
GA	Glyceraldehyde
GO	Glyoxal
LD50	Lethal dose 50
MG	Methylglyoxal
MSCs	Mesenchymal stem cells
RAGE	Receptor for advanced glycation end products
RCS	Reactive carbonyl species
ROS	Reactive oxygen species
TAGE	Toxic AGEs
